# 2,4,6,8-Tetra­kis(2-fluoro­phen­yl)-3,7-diaza­bicyclo­[3.3.1]nonan-9-one

**DOI:** 10.1107/S1600536812051574

**Published:** 2013-01-04

**Authors:** Dong Ho Park, V. Ramkumar, P. Parthiban

**Affiliations:** a Department of Biomedicinal Chemistry, Inje University, Gimhae, Gyeongnam 621 749, Republic of Korea; b Department of Chemistry, IIT Madras, Chennai 600 036, TamilNadu, India

## Abstract

The title compound, C_31_H_24_F_4_N_2_O, exists in a chair–boat conformation with an equatorial orientation of the 2-fluoro­phenyl groups on both sides of the secondary amino group of the chair form. The benzene rings in the ‘chair’ part are inclined to each other at 19.4 (1)°, while the equivalent angle between the benzene rings in the ‘boat’ part is 75.6 (1)°. One F atom was treated as disordered over two positions in a 0.838 (4):0.162 (4) ratio. In the crystal, N—H⋯O hydrogen bonds link the mol­ecules into chains along [001] and these chains are held together *via* weak N—H⋯F and C—H⋯F inter­actions.

## Related literature

For the synthesis and stereochemistry of 3,7-diaza­bicyclo­[3.3.1]nonan-9-ones, see: Parthiban *et al.* (2008 ▶). For the biological activity of 3,7-diaza­bicyclo­[3.3.1]nonan-9-one derivatives and related structures, see: Park *et al.* (2012 ▶); Parthiban *et al.* (2009 ▶, 2010 ▶); Asakawa (1995 ▶); Jeyaraman & Avila (1981 ▶). For ring puckering parameters, see: Cremer & Pople (1975 ▶).
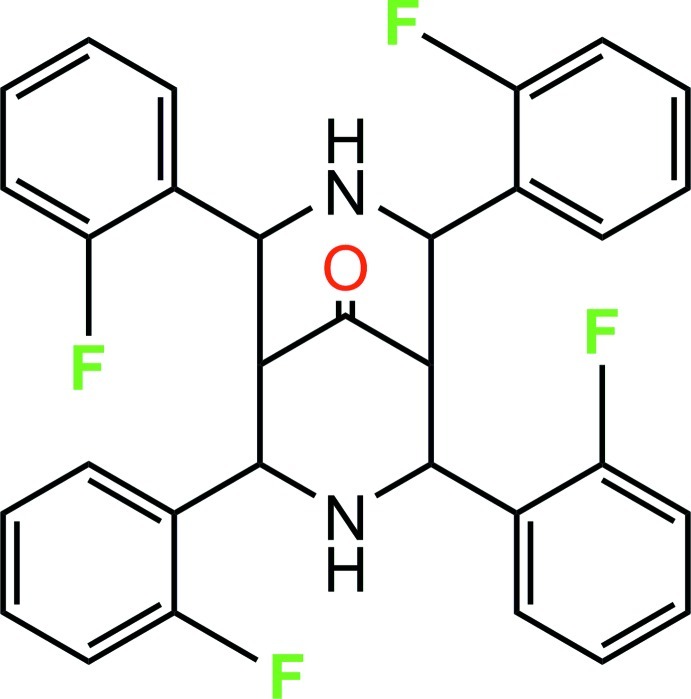


## Experimental

### 

#### Crystal data

C_31_H_24_F_4_N_2_O
*M* *_r_* = 516.52
Monoclinic, 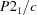

*a* = 12.5610 (11) Å
*b* = 15.9118 (13) Å
*c* = 13.0221 (8) Å
β = 103.207 (3)°
*V* = 2533.9 (3) Å^3^
*Z* = 4
Mo *K*α radiation
μ = 0.10 mm^−1^
*T* = 298 K
0.45 × 0.35 × 0.22 mm

#### Data collection

Bruker APEXII CCD area-detector diffractometer
Absorption correction: multi-scan (*SADABS*; Bruker, 2004 ▶) *T*_min_ = 0.955, *T*_max_ = 0.978
18116 measured reflections
5466 independent reflections
3571 reflections with *I* > 2σ(*I*)
*R*_int_ = 0.024

#### Refinement

*R*[*F* ^2^ > 2σ(*F* ^2^)] = 0.053
*wR*(*F* ^2^) = 0.163
*S* = 1.05
5466 reflections
361 parameters
2 restraints
H atoms treated by a mixture of independent and constrained refinement
Δρ_max_ = 0.48 e Å^−3^
Δρ_min_ = −0.40 e Å^−3^

### 

Data collection: *APEX2* (Bruker, 2004 ▶); cell refinement: *SAINT* (Bruker, 2004 ▶); data reduction: *SAINT*; program(s) used to solve structure: *SHELXS97* (Sheldrick, 2008 ▶); program(s) used to refine structure: *SHELXL97* (Sheldrick, 2008 ▶); molecular graphics: *ORTEP-3* (Farrugia, 2012 ▶); software used to prepare material for publication: *SHELXL97*.

## Supplementary Material

Crystal structure: contains datablock(s) global, I. DOI: 10.1107/S1600536812051574/cv5368sup1.cif

Structure factors: contains datablock(s) I. DOI: 10.1107/S1600536812051574/cv5368Isup2.hkl

Supplementary material file. DOI: 10.1107/S1600536812051574/cv5368Isup3.cml

Additional supplementary materials:  crystallographic information; 3D view; checkCIF report

## Figures and Tables

**Table 1:** Hydrogen-bond geometry (Å, °)

*D*—H⋯*A*	*D*—H	H⋯*A*	*D*⋯*A*	*D*—H⋯*A*
N1—H1*N*⋯O1^i^	0.86 (2)	2.26 (2)	3.119 (2)	175.2 (19)
N2—H2*N*⋯F1^ii^	0.92 (2)	2.46 (2)	3.332 (2)	158.4 (17)
C22—H22⋯F4^iii^	0.93	2.52	3.345 (3)	148

Symmetry codes: (i) 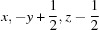; (ii) 
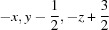
; (iii) 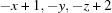.

**Fig. 1.**
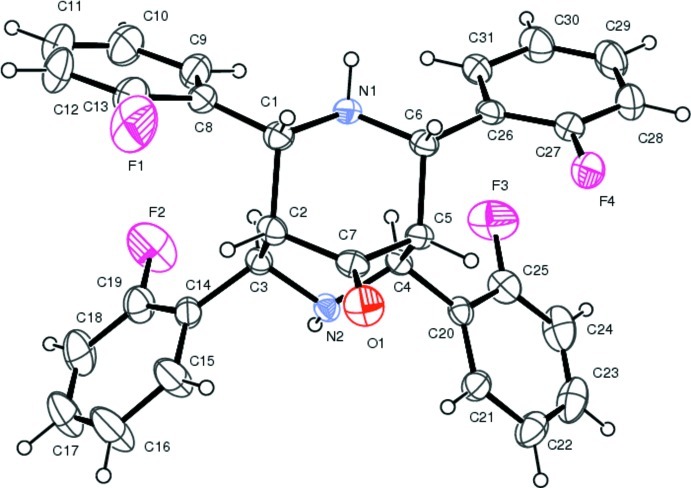
View of (I) showing the atomic numbering and 30% probability displacement ellipsoids. For the disordered atoms, only major part is shown.

## supplementary crystallographic information

### Comment

The Lupin alkaloids contain the 3,7-diazabicyclo[3.3.1]nonan-9-one nucleus, displaying various biological actions (Parthiban *et al.*, 2009, 2010; Asakawa, 1995; Jeyaraman & Avila, 1981). Since the biological actions mainly depend on the stereochemistry of the molecules, we examined the title compound, (I), to explore its stereochemistry in the solid-state.

In (I) (Fig. 1), the piperidone ring N1—C1—C2—C7—C5—C6 adopts a chair conformation, according to Cremer & Pople (1975). The total puckering amplitude Q_T_ is 0.623 (2) Å, and the phase angle θ is 2.7 (3)°. The piperidone ring N2—C3—C2—C7—C5—C4 adopts a boat conformation with Q_T_ = 0.799 (2) and θ = 90.38 (14)°. The 2-fluorophenyl groups attached to the 'chair' piperidone ring are in equatoril position with the torsion angles C7—C2—C1—C8 = 179.04 (19)° and C26—C6—C5—C7 = 176.67 (19)°. The 2-fluorophenyl groups attached to the 'boat' piperidone ring have the following torsion angles - C7—C2—C3—C14 = 116.8 (2)° and C20—C4—C5—C7 = -122.5 (2)°. The benzene rings of the 2-fluorophenyl group on the chair form piperidone are inclined to each other at an angle of 19.44 (3)°, whereas, the same attached on the boat form are inclined to each other at an angle of 74.55 (5)°. In one of the 2-fluorophenyl groups, the F atom is disordered in two positions in a ratio 0.838 (4):0.162 (4). On the basis of the above analysis, it is concluded that the title compound exists in the chair-boat conformation with an equatorial orientation of the 2-fluorophenyl groups on both sides of the secondary amino group of the piperidone in the chair conformation.

In the crystal, intermolecular N—H···O hydrogen bonds (Table 1) link the molecules into chains in [001], and these chains held together *via* weak N—H···F and C—H···F interactions (Table 1).

### Experimental

The 2,4,6,8-*tetrakis*(2-fluorophenyl)-3,7-diazabicyclo[3.3.1] nonan-9-one was synthesized by successive Mannich condensations in one-pot, using 2-fluorobenzaldehyde (0.2 mol, 21 ml), acetone (0.05 mol, 3.7 ml) and ammonium acetate (0.1 mol, 7.7 g) in a 50 ml of absolute ethanol (Parthiban *et al.*, 2008). The mixture was gently warmed on a hot plate at 303 K (30° C) with moderate stirring till the complete consumption of the starting materials, which was monitored by TLC. At the end, the crude 3,7-diazabicycle was separated by filtration and gently washed with 1:5 cold ethanol-ether mixture. The X-ray diffraction quality crystals of pure 2,4,6,8- *tetrakis*(2-fluorophenyl)-3,7-diazabicyclo[3.3.1]nonan-9-one was obtained by slow evaporation from ethanol.

### Refinement

N-bound H atoms were located in a difference Fourier map and refined isotropically. Other hydrogen atoms were fixed geometrically and allowed to ride on the parent carbon atoms with aromatic C—H = 0.93 Å, aliphatic C—H = 0.98 Å and methylene C—H = 0.97 Å. The displacement parameters were set for phenyl, methylene and aliphatic H atoms at *U*_iso_(H) = 1.2*U*_eq_(C) and for methyl H atoms at *U*_iso_(H) = 1.5*U*_eq_(C). In one of the 4-fluorophenyl group the F atom is disordered over two positions in a ratio 0.838 (4):0.162 (4).

### Crystal data

**Table:**

C_31_H_24_F_4_N_2_O	*F*(000) = 1072
*M**_r_* = 516.52	*D*_x_ = 1.354 Mg m^−^^3^
Monoclinic, *P*2_1_/*c*	Mo *K*α radiation, λ = 0.71073 Å
Hall symbol: -P 2ybc	Cell parameters from 5770 reflections
*a* = 12.5610 (11) Å	θ = 2.9–25.5°
*b* = 15.9118 (13) Å	µ = 0.10 mm^−^^1^
*c* = 13.0221 (8) Å	*T* = 298 K
β = 103.207 (3)°	Rectangular, colourless
*V* = 2533.9 (3) Å^3^	0.45 × 0.35 × 0.22 mm
*Z* = 4	

### Data collection

**Table:**

Bruker APEXII CCD area-detector diffractometer	5466 independent reflections
Radiation source: fine-focus sealed tube	3571 reflections with *I* > 2σ(*I*)
Graphite monochromator	*R*_int_ = 0.024
phi and ω scans	θ_max_ = 28.6°, θ_min_ = 2.1°
Absorption correction: multi-scan (*SADABS*; Bruker, 2004)	*h* = −12→16
*T*_min_ = 0.955, *T*_max_ = 0.978	*k* = −21→20
18116 measured reflections	*l* = −17→13

### Refinement

**Table:**

Refinement on *F*^2^	Primary atom site location: structure-invariant direct methods
Least-squares matrix: full	Secondary atom site location: difference Fourier map
*R*[*F*^2^ > 2σ(*F*^2^)] = 0.053	Hydrogen site location: inferred from neighbouring sites
*wR*(*F*^2^) = 0.163	H atoms treated by a mixture of independent and constrained refinement
*S* = 1.05	*w* = 1/[σ^2^(*F*_o_^2^) + (0.0766*P*)^2^ + 0.6943*P*] where *P* = (*F*_o_^2^ + 2*F*_c_^2^)/3
5466 reflections	(Δ/σ)_max_ < 0.001
361 parameters	Δρ_max_ = 0.48 e Å^−^^3^
2 restraints	Δρ_min_ = −0.40 e Å^−^^3^

### Special details

**Table:**

Geometry. All e.s.d.'s (except the e.s.d. in the dihedral angle between two l.s. planes)are estimated using the full covariance matrix. The cell e.s.d.'s are takeninto account individually in the estimation of e.s.d.'s in distances, anglesand torsion angles; correlations between e.s.d.'s in cell parameters are onlyused when they are defined by crystal symmetry. An approximate (isotropic)treatment of cell e.s.d.'s is used for estimating e.s.d.'s involving l.s. planes.
Refinement. Refinement of *F*^2^ against ALL reflections. The weighted *R*-factor *wR* andgoodness of fit *S* are based on *F*^2^, conventional *R*-factors *R* are basedon *F*, with *F* set to zero for negative *F*^2^. The threshold expression of*F*^2^ > σ(*F*^2^) is used only for calculating *R*-factors(gt) *etc*. and isnot relevant to the choice of reflections for refinement. *R*-factors basedon *F*^2^ are statistically about twice as large as those based on *F*, and *R*-factors based on ALL data will be even larger.

### Fractional atomic coordinates and isotropic or equivalent isotropic displacement parameters (Å^2^)

**Table:**

	*x*	*y*	*z*	*U*_iso_*/*U*_eq_	Occ. (<1)
C1	0.09684 (16)	0.22644 (12)	0.67314 (15)	0.0421 (5)	
H1	0.1138	0.2839	0.6990	0.051*	
C2	0.08454 (15)	0.16959 (12)	0.76747 (14)	0.0396 (4)	
H2	0.0282	0.1930	0.8003	0.048*	
C3	0.05623 (15)	0.07695 (11)	0.73535 (14)	0.0374 (4)	
H3	0.0472	0.0708	0.6590	0.045*	
C4	0.25113 (15)	0.04433 (11)	0.76060 (13)	0.0349 (4)	
H4	0.2405	0.0412	0.6838	0.042*	
C5	0.28201 (15)	0.13566 (11)	0.79861 (13)	0.0352 (4)	
H5	0.3511	0.1355	0.8519	0.042*	
C6	0.29129 (15)	0.19517 (11)	0.70649 (14)	0.0374 (4)	
H6	0.3062	0.2522	0.7344	0.045*	
C7	0.19273 (16)	0.17098 (11)	0.84505 (14)	0.0399 (4)	
C8	−0.00906 (17)	0.22752 (13)	0.58965 (17)	0.0486 (5)	
C9	−0.02763 (19)	0.18027 (17)	0.49859 (18)	0.0627 (6)	
H9	0.0292	0.1488	0.4834	0.075*	
C10	−0.1295 (2)	0.1787 (2)	0.4290 (2)	0.0863 (9)	
H10	−0.1399	0.1463	0.3681	0.104*	
C11	−0.2141 (3)	0.2240 (3)	0.4492 (3)	0.0970 (11)	
H11	−0.2824	0.2219	0.4028	0.116*	
C12	−0.1985 (2)	0.2725 (2)	0.5373 (3)	0.0974 (11)	
H12	−0.2554	0.3045	0.5516	0.117*	
C13	−0.0969 (2)	0.27338 (16)	0.6050 (2)	0.0709 (7)	
C14	−0.04489 (16)	0.04573 (13)	0.76692 (16)	0.0445 (5)	
C15	−0.0597 (2)	0.0564 (2)	0.86796 (19)	0.0782 (8)	
H15	−0.0088	0.0877	0.9160	0.094*	
C16	−0.1475 (3)	0.0222 (3)	0.8997 (3)	0.1031 (12)	
H16	−0.1546	0.0297	0.9687	0.124*	
C17	−0.2242 (2)	−0.0226 (2)	0.8300 (3)	0.0946 (10)	
H17	−0.2842	−0.0449	0.8511	0.113*	
C18	−0.2129 (2)	−0.0345 (2)	0.7304 (3)	0.0840 (9)	
H18	−0.2647	−0.0652	0.6825	0.101*	
C19	−0.1239 (2)	−0.00066 (16)	0.70106 (19)	0.0620 (6)	
C20	0.33704 (15)	−0.01785 (11)	0.81200 (15)	0.0390 (4)	
C22	0.4343 (2)	−0.09151 (15)	0.9675 (2)	0.0670 (7)	
H22	0.4433	−0.1027	1.0391	0.080*	
C23	0.4977 (2)	−0.13167 (15)	0.9108 (3)	0.0787 (9)	
H23	0.5507	−0.1698	0.9436	0.094*	
C24	0.4831 (2)	−0.11565 (16)	0.8054 (3)	0.0785 (8)	
H24	0.5269	−0.1419	0.7662	0.094*	
C26	0.38407 (15)	0.16825 (11)	0.65687 (14)	0.0396 (4)	
C27	0.49103 (17)	0.17460 (13)	0.71274 (16)	0.0470 (5)	
C28	0.57966 (19)	0.15100 (16)	0.6745 (2)	0.0643 (6)	
H28	0.6503	0.1554	0.7159	0.077*	
C29	0.5608 (2)	0.12072 (17)	0.5734 (2)	0.0706 (7)	
H29	0.6194	0.1053	0.5449	0.085*	
C30	0.4567 (2)	0.11314 (17)	0.5146 (2)	0.0656 (6)	
H30	0.4446	0.0920	0.4464	0.079*	
C31	0.36851 (18)	0.13667 (13)	0.55559 (16)	0.0501 (5)	
H31	0.2979	0.1311	0.5144	0.060*	
F1	−0.08125 (15)	0.32238 (11)	0.69330 (16)	0.1080 (6)	
F2	−0.11291 (17)	−0.01465 (15)	0.60175 (14)	0.1208 (8)	
F4	0.50996 (10)	0.20708 (9)	0.81229 (10)	0.0628 (4)	
N1	0.18692 (13)	0.19524 (10)	0.63013 (13)	0.0407 (4)	
H1N	0.1914 (17)	0.2264 (13)	0.5771 (17)	0.047 (6)*	
N2	0.14804 (12)	0.02386 (10)	0.79069 (12)	0.0384 (4)	
H2N	0.1312 (17)	−0.0318 (15)	0.7762 (15)	0.050 (6)*	
O1	0.20898 (13)	0.20153 (11)	0.93241 (11)	0.0625 (4)	
C21	0.35739 (17)	−0.03465 (13)	0.91879 (17)	0.0520 (5)	
H21	0.3171	−0.0061	0.9594	0.062*	0.838 (4)
F3A	0.3015 (5)	0.0083 (4)	0.9768 (5)	0.082 (4)	0.162 (4)
C25	0.40231 (19)	−0.06003 (13)	0.75767 (19)	0.0565 (6)	
H21A	0.3917	−0.0507	0.6855	0.068*	0.162 (4)
F3	0.38896 (17)	−0.04757 (12)	0.65546 (14)	0.0842 (8)	0.838 (4)

### Atomic displacement parameters (Å^2^)

**Table:**

	*U*^11^	*U*^22^	*U*^33^	*U*^12^	*U*^13^	*U*^23^
C1	0.0463 (12)	0.0347 (10)	0.0476 (11)	0.0057 (8)	0.0154 (9)	0.0037 (8)
C2	0.0432 (11)	0.0402 (10)	0.0402 (9)	0.0048 (8)	0.0195 (8)	−0.0030 (8)
C3	0.0368 (10)	0.0416 (10)	0.0351 (9)	0.0020 (8)	0.0110 (8)	−0.0013 (7)
C4	0.0377 (10)	0.0348 (9)	0.0342 (9)	−0.0026 (7)	0.0123 (8)	−0.0016 (7)
C5	0.0386 (11)	0.0348 (9)	0.0324 (8)	−0.0029 (7)	0.0084 (8)	0.0012 (7)
C6	0.0406 (11)	0.0327 (9)	0.0404 (9)	−0.0012 (8)	0.0126 (8)	0.0032 (8)
C7	0.0518 (12)	0.0342 (9)	0.0371 (9)	−0.0029 (8)	0.0169 (9)	−0.0018 (8)
C8	0.0435 (12)	0.0481 (11)	0.0574 (12)	0.0114 (9)	0.0178 (10)	0.0172 (10)
C9	0.0527 (14)	0.0828 (17)	0.0510 (12)	0.0133 (12)	0.0084 (11)	0.0049 (12)
C10	0.0658 (19)	0.121 (3)	0.0641 (16)	0.0033 (18)	−0.0028 (14)	0.0096 (16)
C11	0.0544 (19)	0.126 (3)	0.101 (2)	0.0119 (18)	−0.0019 (17)	0.035 (2)
C12	0.0531 (18)	0.102 (2)	0.137 (3)	0.0391 (16)	0.0203 (19)	0.038 (2)
C13	0.0689 (18)	0.0603 (15)	0.0879 (18)	0.0262 (13)	0.0271 (15)	0.0130 (14)
C14	0.0338 (11)	0.0501 (11)	0.0490 (11)	0.0006 (9)	0.0086 (9)	0.0004 (9)
C15	0.0657 (17)	0.117 (2)	0.0589 (14)	−0.0358 (16)	0.0284 (13)	−0.0164 (15)
C16	0.085 (2)	0.154 (3)	0.087 (2)	−0.048 (2)	0.0537 (18)	−0.024 (2)
C17	0.0587 (18)	0.118 (3)	0.118 (3)	−0.0284 (17)	0.0429 (18)	−0.008 (2)
C18	0.0473 (16)	0.099 (2)	0.103 (2)	−0.0296 (14)	0.0103 (15)	−0.0190 (17)
C19	0.0531 (15)	0.0736 (16)	0.0579 (14)	−0.0078 (12)	0.0097 (11)	−0.0140 (12)
C20	0.0347 (10)	0.0317 (9)	0.0517 (11)	−0.0046 (7)	0.0121 (9)	−0.0001 (8)
C22	0.0588 (16)	0.0522 (14)	0.0775 (16)	−0.0064 (12)	−0.0104 (13)	0.0168 (12)
C23	0.0567 (17)	0.0413 (13)	0.123 (3)	0.0055 (11)	−0.0104 (16)	0.0058 (15)
C24	0.0616 (17)	0.0480 (14)	0.130 (3)	0.0146 (12)	0.0307 (17)	−0.0136 (16)
C26	0.0418 (11)	0.0355 (10)	0.0441 (10)	−0.0014 (8)	0.0149 (9)	0.0092 (8)
C27	0.0459 (13)	0.0471 (11)	0.0482 (11)	−0.0043 (9)	0.0109 (9)	0.0076 (9)
C28	0.0394 (13)	0.0721 (16)	0.0820 (17)	0.0034 (11)	0.0151 (12)	0.0116 (13)
C29	0.0545 (16)	0.0823 (18)	0.0846 (18)	0.0079 (13)	0.0359 (14)	−0.0012 (15)
C30	0.0631 (17)	0.0791 (17)	0.0621 (14)	0.0056 (13)	0.0299 (12)	−0.0059 (12)
C31	0.0462 (12)	0.0595 (13)	0.0476 (11)	0.0002 (10)	0.0167 (9)	0.0028 (10)
F1	0.1086 (14)	0.0917 (12)	0.1292 (15)	0.0536 (11)	0.0388 (11)	−0.0123 (11)
F2	0.1206 (16)	0.1637 (19)	0.0805 (11)	−0.0621 (14)	0.0277 (11)	−0.0527 (12)
F4	0.0538 (8)	0.0760 (9)	0.0545 (8)	−0.0110 (6)	0.0040 (6)	0.0014 (6)
N1	0.0402 (10)	0.0455 (9)	0.0387 (8)	0.0055 (7)	0.0140 (7)	0.0106 (7)
N2	0.0350 (9)	0.0349 (9)	0.0468 (9)	−0.0021 (7)	0.0123 (7)	0.0028 (7)
O1	0.0697 (11)	0.0749 (11)	0.0451 (8)	−0.0027 (8)	0.0179 (7)	−0.0241 (7)
C21	0.0460 (13)	0.0501 (12)	0.0568 (13)	−0.0031 (10)	0.0054 (10)	0.0101 (10)
F3A	0.092 (8)	0.107 (8)	0.047 (5)	0.011 (6)	0.015 (5)	0.019 (5)
C25	0.0596 (14)	0.0415 (11)	0.0733 (15)	0.0040 (10)	0.0254 (12)	−0.0051 (10)
F3	0.1167 (17)	0.0793 (13)	0.0713 (12)	0.0305 (11)	0.0522 (11)	−0.0031 (9)

### Geometric parameters (Å, º)

**Table:**

C1—N1	1.459 (2)	C15—H15	0.9300
C1—C8	1.514 (3)	C16—C17	1.364 (4)
C1—C2	1.561 (3)	C16—H16	0.9300
C1—H1	0.9800	C17—C18	1.350 (4)
C2—C7	1.497 (3)	C17—H17	0.9300
C2—C3	1.551 (3)	C18—C19	1.373 (4)
C2—H2	0.9800	C18—H18	0.9300
C3—N2	1.478 (2)	C19—F2	1.350 (3)
C3—C14	1.506 (3)	C20—C25	1.374 (3)
C3—H3	0.9800	C20—C21	1.381 (3)
C4—N2	1.473 (2)	C22—C23	1.363 (4)
C4—C20	1.504 (3)	C22—C21	1.369 (3)
C4—C5	1.555 (2)	C22—H22	0.9300
C4—H4	0.9800	C23—C24	1.366 (4)
C5—C7	1.500 (3)	C23—H23	0.9300
C5—C6	1.553 (2)	C24—C25	1.382 (3)
C5—H5	0.9800	C24—H24	0.9300
C6—N1	1.453 (2)	C26—C27	1.378 (3)
C6—C26	1.518 (3)	C26—C31	1.383 (3)
C6—H6	0.9800	C27—F4	1.365 (2)
C7—O1	1.211 (2)	C27—C28	1.371 (3)
C8—C13	1.375 (3)	C28—C29	1.371 (4)
C8—C9	1.378 (3)	C28—H28	0.9300
C9—C10	1.389 (3)	C29—C30	1.362 (3)
C9—H9	0.9300	C29—H29	0.9300
C10—C11	1.358 (4)	C30—C31	1.386 (3)
C10—H10	0.9300	C30—H30	0.9300
C11—C12	1.360 (5)	C31—H31	0.9300
C11—H11	0.9300	N1—H1N	0.86 (2)
C12—C13	1.376 (4)	N2—H2N	0.92 (2)
C12—H12	0.9300	C21—F3A	1.331 (2)
C13—F1	1.366 (3)	C21—H21	0.9300
C14—C19	1.370 (3)	C25—F3	1.318 (3)
C14—C15	1.381 (3)	C25—H21A	0.9300
C15—C16	1.376 (3)		
			
N1—C1—C8	111.06 (16)	C14—C15—H15	119.0
N1—C1—C2	109.24 (15)	C17—C16—C15	120.0 (3)
C8—C1—C2	110.06 (16)	C17—C16—H16	120.0
N1—C1—H1	108.8	C15—C16—H16	120.0
C8—C1—H1	108.8	C18—C17—C16	120.0 (3)
C2—C1—H1	108.8	C18—C17—H17	120.0
C7—C2—C3	108.22 (15)	C16—C17—H17	120.0
C7—C2—C1	106.54 (15)	C17—C18—C19	118.9 (3)
C3—C2—C1	113.34 (14)	C17—C18—H18	120.5
C7—C2—H2	109.5	C19—C18—H18	120.5
C3—C2—H2	109.5	F2—C19—C14	118.1 (2)
C1—C2—H2	109.5	F2—C19—C18	118.0 (2)
N2—C3—C14	106.93 (15)	C14—C19—C18	123.9 (2)
N2—C3—C2	107.85 (15)	C25—C20—C21	115.4 (2)
C14—C3—C2	113.30 (15)	C25—C20—C4	122.96 (18)
N2—C3—H3	109.6	C21—C20—C4	121.63 (17)
C14—C3—H3	109.6	C23—C22—C21	119.8 (3)
C2—C3—H3	109.6	C23—C22—H22	120.1
N2—C4—C20	108.73 (14)	C21—C22—H22	120.1
N2—C4—C5	107.01 (14)	C22—C23—C24	119.7 (2)
C20—C4—C5	111.82 (15)	C22—C23—H23	120.1
N2—C4—H4	109.7	C24—C23—H23	120.1
C20—C4—H4	109.7	C23—C24—C25	119.2 (2)
C5—C4—H4	109.7	C23—C24—H24	120.4
C7—C5—C6	106.14 (14)	C25—C24—H24	120.4
C7—C5—C4	108.87 (14)	C27—C26—C31	116.01 (18)
C6—C5—C4	112.39 (14)	C27—C26—C6	120.37 (17)
C7—C5—H5	109.8	C31—C26—C6	123.62 (17)
C6—C5—H5	109.8	F4—C27—C28	117.94 (19)
C4—C5—H5	109.8	F4—C27—C26	117.80 (17)
N1—C6—C26	111.68 (15)	C28—C27—C26	124.3 (2)
N1—C6—C5	108.28 (15)	C29—C28—C27	117.9 (2)
C26—C6—C5	110.93 (15)	C29—C28—H28	121.0
N1—C6—H6	108.6	C27—C28—H28	121.0
C26—C6—H6	108.6	C30—C29—C28	120.3 (2)
C5—C6—H6	108.6	C30—C29—H29	119.9
O1—C7—C2	124.89 (17)	C28—C29—H29	119.9
O1—C7—C5	123.25 (18)	C29—C30—C31	120.6 (2)
C2—C7—C5	111.70 (15)	C29—C30—H30	119.7
C13—C8—C9	115.5 (2)	C31—C30—H30	119.7
C13—C8—C1	120.3 (2)	C26—C31—C30	120.9 (2)
C9—C8—C1	124.06 (18)	C26—C31—H31	119.5
C8—C9—C10	121.4 (2)	C30—C31—H31	119.5
C8—C9—H9	119.3	C6—N1—C1	113.27 (15)
C10—C9—H9	119.3	C6—N1—H1N	109.6 (14)
C11—C10—C9	120.6 (3)	C1—N1—H1N	108.1 (14)
C11—C10—H10	119.7	C4—N2—C3	112.30 (14)
C9—C10—H10	119.7	C4—N2—H2N	109.5 (13)
C10—C11—C12	119.7 (3)	C3—N2—H2N	109.4 (13)
C10—C11—H11	120.1	F3A—C21—C22	119.0 (4)
C12—C11—H11	120.1	F3A—C21—C20	118.1 (4)
C11—C12—C13	118.7 (3)	C22—C21—C20	122.9 (2)
C11—C12—H12	120.6	F3A—C21—H21	1.7
C13—C12—H12	120.6	C22—C21—H21	118.6
F1—C13—C8	117.4 (2)	C20—C21—H21	118.6
F1—C13—C12	118.6 (3)	F3—C25—C20	119.6 (2)
C8—C13—C12	124.0 (3)	F3—C25—C24	117.5 (2)
C19—C14—C15	115.3 (2)	C20—C25—C24	123.0 (2)
C19—C14—C3	122.84 (18)	F3—C25—H21A	1.1
C15—C14—C3	121.68 (18)	C20—C25—H21A	118.5
C16—C15—C14	122.0 (2)	C24—C25—H21A	118.5
C16—C15—H15	119.0		
			
N1—C1—C2—C7	56.93 (19)	C15—C14—C19—F2	−178.8 (3)
C8—C1—C2—C7	179.12 (15)	C3—C14—C19—F2	−3.8 (3)
N1—C1—C2—C3	−62.0 (2)	C15—C14—C19—C18	0.5 (4)
C8—C1—C2—C3	60.2 (2)	C3—C14—C19—C18	175.5 (2)
C7—C2—C3—N2	−1.65 (18)	C17—C18—C19—F2	178.7 (3)
C1—C2—C3—N2	116.29 (16)	C17—C18—C19—C14	−0.5 (5)
C7—C2—C3—C14	116.50 (16)	N2—C4—C20—C25	130.75 (19)
C1—C2—C3—C14	−125.56 (17)	C5—C4—C20—C25	−111.3 (2)
N2—C4—C5—C7	−3.65 (19)	N2—C4—C20—C21	−51.0 (2)
C20—C4—C5—C7	−122.62 (16)	C5—C4—C20—C21	66.9 (2)
N2—C4—C5—C6	−120.95 (15)	C21—C22—C23—C24	−0.8 (4)
C20—C4—C5—C6	120.09 (16)	C22—C23—C24—C25	−1.3 (4)
C7—C5—C6—N1	−60.26 (18)	N1—C6—C26—C27	171.53 (17)
C4—C5—C6—N1	58.65 (19)	C5—C6—C26—C27	−67.6 (2)
C7—C5—C6—C26	176.86 (15)	N1—C6—C26—C31	−8.4 (2)
C4—C5—C6—C26	−64.2 (2)	C5—C6—C26—C31	112.5 (2)
C3—C2—C7—O1	−123.9 (2)	C31—C26—C27—F4	178.71 (17)
C1—C2—C7—O1	113.9 (2)	C6—C26—C27—F4	−1.2 (3)
C3—C2—C7—C5	60.54 (18)	C31—C26—C27—C28	−0.8 (3)
C1—C2—C7—C5	−61.65 (18)	C6—C26—C27—C28	179.30 (19)
C6—C5—C7—O1	−112.1 (2)	F4—C27—C28—C29	−178.1 (2)
C4—C5—C7—O1	126.71 (19)	C26—C27—C28—C29	1.4 (4)
C6—C5—C7—C2	63.50 (18)	C27—C28—C29—C30	−1.3 (4)
C4—C5—C7—C2	−57.69 (18)	C28—C29—C30—C31	0.7 (4)
N1—C1—C8—C13	−163.67 (19)	C27—C26—C31—C30	0.1 (3)
C2—C1—C8—C13	75.2 (2)	C6—C26—C31—C30	180.0 (2)
N1—C1—C8—C9	20.7 (3)	C29—C30—C31—C26	0.0 (4)
C2—C1—C8—C9	−100.4 (2)	C26—C6—N1—C1	−175.95 (15)
C13—C8—C9—C10	−1.2 (4)	C5—C6—N1—C1	61.62 (19)
C1—C8—C9—C10	174.7 (2)	C8—C1—N1—C6	178.37 (15)
C8—C9—C10—C11	−0.1 (4)	C2—C1—N1—C6	−60.0 (2)
C9—C10—C11—C12	1.1 (5)	C20—C4—N2—C3	−174.49 (14)
C10—C11—C12—C13	−0.9 (5)	C5—C4—N2—C3	64.57 (18)
C9—C8—C13—F1	−178.7 (2)	C14—C3—N2—C4	176.03 (14)
C1—C8—C13—F1	5.3 (3)	C2—C3—N2—C4	−61.80 (18)
C9—C8—C13—C12	1.4 (4)	C23—C22—C21—F3A	−175.4 (4)
C1—C8—C13—C12	−174.6 (2)	C23—C22—C21—C20	2.7 (3)
C11—C12—C13—F1	179.7 (3)	C25—C20—C21—F3A	175.8 (3)
C11—C12—C13—C8	−0.4 (5)	C4—C20—C21—F3A	−2.5 (3)
N2—C3—C14—C19	−105.3 (2)	C25—C20—C21—C22	−2.3 (3)
C2—C3—C14—C19	136.0 (2)	C4—C20—C21—C22	179.37 (18)
N2—C3—C14—C15	69.4 (3)	C21—C20—C25—F3	−179.71 (19)
C2—C3—C14—C15	−49.3 (3)	C4—C20—C25—F3	−1.4 (3)
C19—C14—C15—C16	0.4 (4)	C21—C20—C25—C24	0.0 (3)
C3—C14—C15—C16	−174.7 (3)	C4—C20—C25—C24	178.4 (2)
C14—C15—C16—C17	−1.2 (6)	C23—C24—C25—F3	−178.5 (2)
C15—C16—C17—C18	1.1 (6)	C23—C24—C25—C20	1.7 (4)
C16—C17—C18—C19	−0.3 (6)		

### Hydrogen-bond geometry (Å, º)

**Table:**

*D*—H···*A*	*D*—H	H···*A*	*D*···*A*	*D*—H···*A*
N1—H1*N*···O1^i^	0.86 (2)	2.26 (2)	3.119 (2)	175.2 (19)
N2—H2*N*···F1^ii^	0.92 (2)	2.46 (2)	3.332 (2)	158.4 (17)
C22—H22···F4^iii^	0.93	2.52	3.345 (3)	148

Symmetry codes: (i) *x*, −*y*+1/2, *z*−1/2; (ii) −*x*, *y*−1/2, −*z*+3/2; (iii) −*x*+1, −*y*, −*z*+2.
